# Crystal structure of 1,1-di­acetyl­ferrocene dihydrazone

**DOI:** 10.1107/S1600536814014366

**Published:** 2014-07-02

**Authors:** Namig G. Shikhaliyev, Atash V. Gurbanov, Vasily M. Muzalevsky, Valentine G. Nenajdenko, Victor N. Khrustalev

**Affiliations:** a Baku State University, Z. Khalilov St 23, Baku, AZ-1148, Azerbaijan; b Chemistry Department, M.V. Lomonosov Moscow State University, Leninskie gory 1/3, Moscow, 119991, Russian Federation; c X-Ray Structural Centre, A.N. Nesmeyanov Institute of Organoelement Compounds, Russian Academy of Sciences, 28 Vavilov St, B-334, Moscow 119991, Russian Federation

**Keywords:** crystal structure, ferrocene, dihydrazone, chiral atropoisomers, hydrogen bonds

## Abstract

The title compound, [Fe(C_7_H_9_N_2_)_2_], crystallizes with two crystallographically independent mol­ecules in the unit cell. These represent the chiral atropoisomers distinguished by the mutual arrangement of the two acet­yl–hydrazone groups with a *cis* conformation of the C=N bonds. The two cyclo­penta­dienyl (Cp) rings are planar and nearly parallel, the tilt between the two rings being 3.16 (16)° [4.40 (18)° for the second independent mol­ecule]. The conformation of the Cp rings is close to eclipsed, the twist angle being 0.1 (2)° [3.3 (2)°]. The two acet­yl–hydrazone substituents are also planar and are inclined at 13.99 (15)/9.17 (16)° [6.83 (17)/14.59 (15)°] relative to the Cp rings. The Fe—C bond lengths range from 2.035 (3) to 2.065 (2) Å, with an average of 2.050 (3) Å [2.036 (3) to 2.069 (2), average 2.046 (3) Å], which agrees well with those reported for most ferrocene derivatives. In the crystal, the mol­ecules form dimers *via* two strong N—H⋯N hydrogen bonds. The dimers are linked into a three-dimensional framework by weak N—H⋯N hydrogen bonds.

## Related literature   

For a new catalytic olefination reaction and synthesis of 1,1-di­acetyl­ferrocene dihydrazone, see: Korotchenko *et al.* (2001 ▶); Nenajdenko *et al.* (2004 ▶); Abd-Elzaher *et al.* (2005 ▶). For related compounds, see: Xiao *et al.* (1999 ▶); Fang *et al.* (2001 ▶); Lopez *et al.* (2003 ▶); Zhang *et al.* (2006 ▶); Zhou *et al.* (2007 ▶); Qiao *et al.* (2009 ▶).
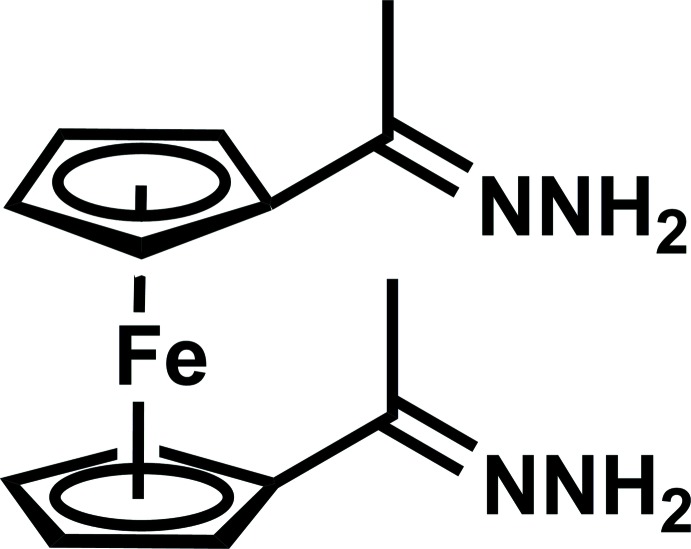


## Experimental   

### Crystal data  

[Fe(C_7_H_9_N_2_)_2_]
*M* *_r_* = 298.17
Orthorhombic, 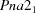

*a* = 9.2647 (3) Å
*b* = 12.9260 (4) Å
*c* = 22.0729 (7) Å
*V* = 2643.35 (15) Å^3^
*Z* = 8
Mo *K*α radiation
μ = 1.13 mm^−1^
*T* = 295 K
0.26 × 0.22 × 0.18 mm

### Data collection  

Bruker APEXII CCD diffractometer
Absorption correction: multi-scan (*SADABS*; Bruker, 2003 ▶) *T*_min_ = 0.757, *T*_max_ = 0.822
38476 measured reflections
6542 independent reflections
6096 reflections with *I* > 2σ(*I*)
*R*_int_ = 0.017

### Refinement  

*R*[*F* ^2^ > 2σ(*F* ^2^)] = 0.031
*wR*(*F* ^2^) = 0.080
*S* = 1.08
6542 reflections
347 parameters
1 restraint
H-atom parameters constrained
Δρ_max_ = 0.43 e Å^−3^
Δρ_min_ = −0.25 e Å^−3^
Absolute structure: Flack (1983 ▶), 3181 Friedel pairs
Absolute structure parameter: 0.475 (16)

### 

Data collection: *APEX2* (Bruker, 2005 ▶); cell refinement: *SAINT* (Bruker, 2001 ▶); data reduction: *SAINT*; program(s) used to solve structure: *SHELXTL* (Sheldrick, 2008 ▶); program(s) used to refine structure: *SHELXTL*; molecular graphics: *SHELXTL*; software used to prepare material for publication: *SHELXTL*.

## Supplementary Material

Crystal structure: contains datablock(s) global, I. DOI: 10.1107/S1600536814014366/rk2429sup1.cif

Structure factors: contains datablock(s) I. DOI: 10.1107/S1600536814014366/rk2429Isup2.hkl

CCDC reference: 1009066

Additional supporting information:  crystallographic information; 3D view; checkCIF report

## Figures and Tables

**Table 1:** Hydrogen-bond geometry (Å, °)

*D*—H⋯*A*	*D*—H	H⋯*A*	*D*⋯*A*	*D*—H⋯*A*
N2—H2*A*⋯N7^i^	0.90	2.53	3.287 (5)	142
N4—H4*A*⋯N5^ii^	0.90	2.29	3.137 (4)	157
N4—H4*B*⋯N2^iii^	0.90	2.61	3.421 (4)	150
N6—H6*A*⋯N3^iv^	0.90	2.24	3.073 (4)	154
N8—H8*B*⋯N1^v^	0.90	2.60	3.497 (5)	178

Symmetry codes: (i) 
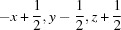
; (ii) 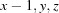; (iii) 
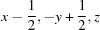
; (iv) 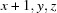; (v) 
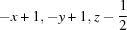
.

**Fig. 1.**
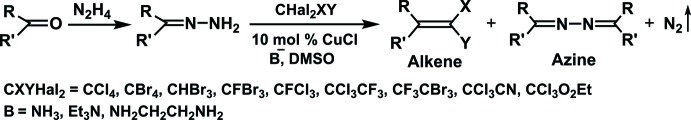
The Nenajdenko–Shastin catalytic olefination reaction as a general method for the preparation of alkenes.

**Fig. 2.**
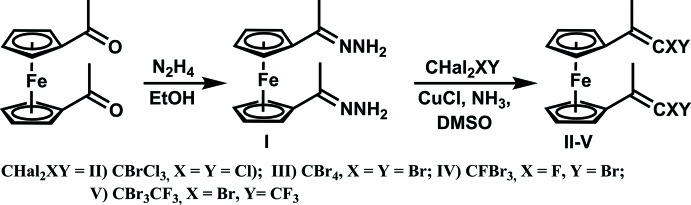
Reaction of 1,1'–diacetylferrocene with hydrazone in C2H5OH.

**Fig. 3.**
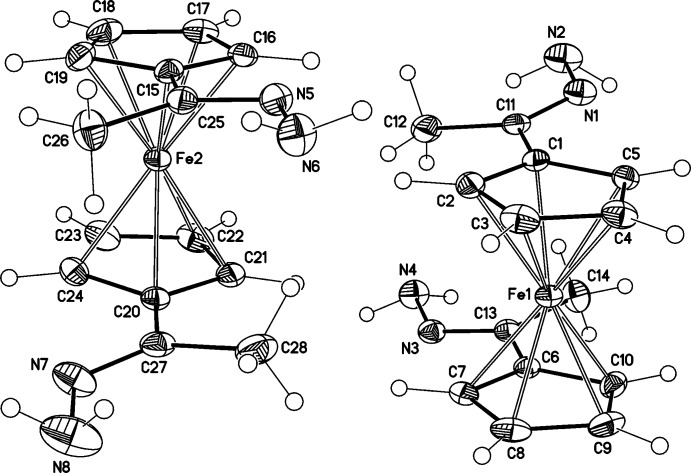
Molecular structure of I with the atom numbering scheme. The two crystallographically independent molecules are presented. Displacement ellipsoids are shown at the 40% probability level. H atoms are depicted as a small spheres of arbitrary radius.

**Fig. 4.**
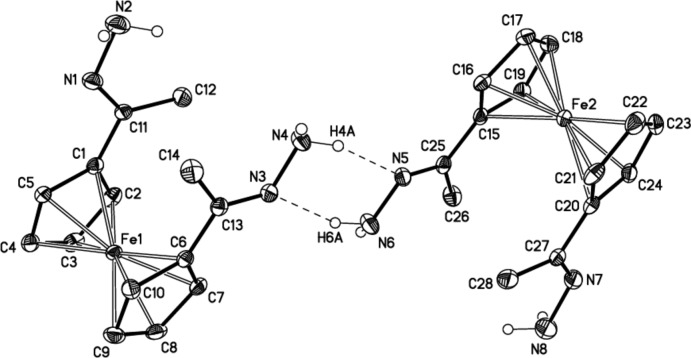
The H–bonded dimers of I. Dashed lines indicate the intermolecular hydrogen bonds.

**Fig. 5.**
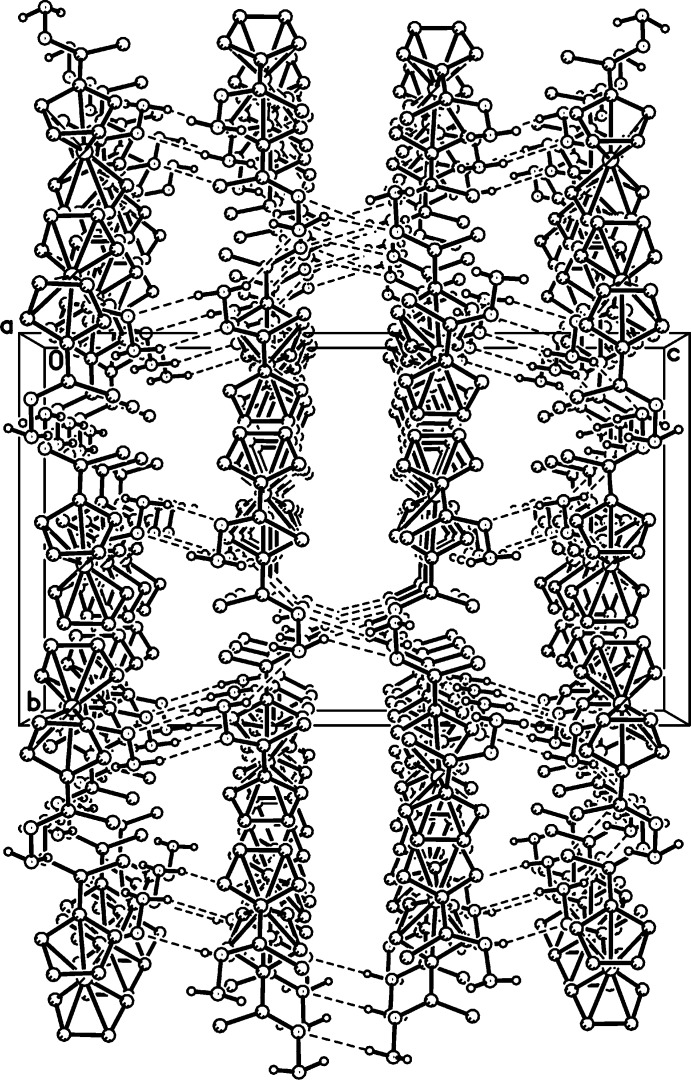
A portion of the crystal packing of I along the a axis. Dashed lines indicate the intermolecular hydrogen bonds.

## supplementary crystallographic information

### S1. Comment

The Nenajdenko–Shastin catalytic olefination reaction discovered by us recently is a facile approach to functionally substituted halogen–alkenes (Korotchenko *et al.*, 2001; Nenajdenko *et al.*, 2004) (Fig. 1). To study the further synthetic potential of this reaction, we have investigated the olefination of 1,1–diacetylferrocene as a representative of metallocenes. The structure of the reaction product, C_14_H_18_FeN_4_, I, has been unambiguously established by X–ray diffraction analysis. It has been revealed that the olefination of 1,1–diacetylferrocene allows the developing of a simple and convenient way to obtain the halogen–substituted ferrocene–alkenes II–V (Fig. 2).

The I crystallizes in the non–centrosymmetric orthorhombic space group *Pna*2_1_ with two crystallographically independent molecules in the unit cell. The two crystallographically independent molecules of I represent the chiral atropoisomers (Fig. 3). The atropoisomers are distinguished by the mutual arrangement of the two acetyl–hydrazone groups having the *cis*–configuration of the C═N bonds (the disposition of the hydrazone groups is right/left and left/right, respectively). The two Cp rings are planar (r.m.s. deviations are 0.001, 0.003 and 0.003, 0.002 for the two crystallographically independent molecules, respectively) and nearly parallel, the ring–tilt between the two rings being 3.16 (16)° and 4.40 (18)° for the two crystallographically independent molecules, respectively. The conformation of the Cp rings is close to eclipsed, which is common for ferrocene derivatives. The twist angle of the Cp rings is defined as the torsion angle between a ring C atom, the two ring centers and the corresponding C atom on the opposite ring. The value of the twist angle in I is 0.1 (2)° and 3.3 (2)° for the two crystallographically independent molecules, respectively. The two acetyl–hydrazone substituents are also planar (r.m.s. deviations are 0.012, 0.026 and 0.018, 0.027 for the two crystallographically independent molecules, respectively) and are inclined relative to the Cp rings at 13.99 (15)°, 9.17 (16)° and 6.83 (17)°, 14.59 (15)° for the two crystallographically independent molecules, respectively. The Fe–C distances are as expected for a ferrocene derivative, ranging from 2.035 (3)Å to 2.065 (2)Å, with an average of 2.050 (3)Å and 2.046 (3)Å (for the two crystallographically independent molecules, respectively), which agree well with those reported for most of ferrocene derivatives (Xiao *et al.*, 1999; Fang *et al.*, 2001; Lopez *et al.*, 2003; Zhang *et al.*, 2006; Zhou *et al.*, 2007; Qiao *et al.*, 2009).

In the crystal, the molecules of I form a dimers *via* the two strong intermolecular N—H···N hydrogen bonds (Fig. 4, Table 1). Further, the dimers are linked into three–dimensional framework by the additional weak intermolecular N—H···N hydrogen bonds (Fig. 5, Table 1).

### S2. Experimental

The product I was prepared by use of the methodics described in Abd-Elzaher *et al.*, 2005. The single crystals of I were obtained by slow crystallization from C_2_H_5_OH. M.p. = 452–454 K.

### S3. Refinement

The value of Flack parameter 0.475 (16) (3181 Friedel pairs measured, 99%) indicates that, in this case, the absolute structure cannot be objectively determined due to the specifical (*pseudo*–centrosymmetrical) arrangement of heavy Fe atoms.

The hydrogen atoms of the amino–groups were localized in the difference Fourier maps and refined isotropically with fixed displacement parameters (*U*_iso_(H) = 1.2*U*_eq_(N)). The other hydrogen atoms were placed in calculated positions with with C—H = 0.96Å (Cp H) and 0.98Å (methyl H) and refined within the riding model with fixed isotropic displacement parameters (*U*_iso_(H) = 1.2*U*_eq_(C)).

### Crystal data

**Table:**

[Fe(C_7_H_9_N_2_)_2_]	*D*_x_ = 1.498 Mg m^−^^3^
*M**_r_* = 298.17	Melting point = 452–454 K
Orthorhombic, *P**n**a*2_1_	Mo *K*α radiation, λ = 0.71073 Å
Hall symbol: P 2c -2n	Cell parameters from 9951 reflections
*a* = 9.2647 (3) Å	θ = 2.7–28.3°
*b* = 12.9260 (4) Å	µ = 1.13 mm^−^^1^
*c* = 22.0729 (7) Å	*T* = 295 K
*V* = 2643.35 (15) Å^3^	Prism, colourless
*Z* = 8	0.26 × 0.22 × 0.18 mm
*F*(000) = 1248	

### Data collection

**Table:**

Bruker APEXII CCD diffractometer	6542 independent reflections
Radiation source: fine–focus sealed tube	6096 reflections with *I* > 2σ(*I*)
Graphite monochromator	*R*_int_ = 0.017
φ and ω scans	θ_max_ = 28.3°, θ_min_ = 1.8°
Absorption correction: multi-scan (*SADABS*; Bruker, 2003)	*h* = −12→12
*T*_min_ = 0.757, *T*_max_ = 0.822	*k* = −17→17
38476 measured reflections	*l* = −29→29

### Refinement

**Table:**

Refinement on *F*^2^	Secondary atom site location: difference Fourier map
Least-squares matrix: full	Hydrogen site location: difference Fourier map
*R*[*F*^2^ > 2σ(*F*^2^)] = 0.031	H-atom parameters constrained
*wR*(*F*^2^) = 0.080	*w* = 1/[σ^2^(*F*_o_^2^) + (0.0361*P*)^2^ + 1.3943*P*] where *P* = (*F*_o_^2^ + 2*F*_c_^2^)/3
*S* = 1.08	(Δ/σ)_max_ = 0.002
6542 reflections	Δρ_max_ = 0.43 e Å^−^^3^
347 parameters	Δρ_min_ = −0.25 e Å^−^^3^
1 restraint	Absolute structure: Flack (1983), 3181 Friedel pairs
Primary atom site location: structure-invariant direct methods	Absolute structure parameter: 0.475 (16)

### Special details

**Table:**

Geometry. All s.u.'s (except the s.u. in the dihedral angle between two l.s. planes) are estimated using the full covariance matrix. The cell s.u.'s are taken into account individually in the estimation of s.u.'s in distances, angles and torsion angles; correlations between s.u.'s in cell parameters are only used when they are defined by crystal symmetry. An approximate (isotropic) treatment of cell s.u.'s is used for estimating s.u.'s involving l.s. planes.
Refinement. Refinement of *F*^2^ against ALL reflections. The weighted *R*–factor *wR* and goodness of fit *S* are based on *F*^2^, conventional *R*–factors *R* are based on *F*, with *F* set to zero for negative *F*^2^. The threshold expression of *F*^2^ > σ(*F*^2^) is used only for calculating *R*–factors(gt) *etc*. and is not relevant to the choice of reflections for refinement. *R*–factors based on *F*^2^ are statistically about twice as large as those based on *F*, and *R*–factors based on ALL data will be even larger.

### Fractional atomic coordinates and isotropic or equivalent isotropic displacement parameters (Å^2^)

**Table:**

	*x*	*y*	*z*	*U*_iso_*/*U*_eq_	
Fe1	0.34263 (3)	0.58615 (2)	0.871244 (14)	0.03239 (8)	
N1	0.2664 (3)	0.29657 (18)	0.92124 (12)	0.0476 (5)	
N2	0.1723 (3)	0.2109 (2)	0.92300 (16)	0.0639 (8)	
H2A	0.1429	0.2401	0.9579	0.077*	
H2B	0.1236	0.2219	0.8883	0.077*	
N3	−0.0469 (3)	0.50928 (18)	0.81874 (11)	0.0417 (5)	
N4	−0.1449 (3)	0.4276 (2)	0.81548 (14)	0.0526 (6)	
H4A	−0.1934	0.4383	0.7807	0.063*	
H4B	−0.2017	0.4161	0.8479	0.063*	
C1	0.3861 (2)	0.42947 (18)	0.87079 (14)	0.0351 (5)	
C2	0.4474 (3)	0.4795 (2)	0.81904 (14)	0.0454 (7)	
H2	0.4260	0.4634	0.7766	0.054*	
C3	0.5449 (3)	0.5561 (3)	0.83974 (16)	0.0500 (7)	
H3	0.6023	0.6021	0.8139	0.060*	
C4	0.5453 (3)	0.5555 (2)	0.90370 (15)	0.0462 (7)	
H4	0.6029	0.6005	0.9300	0.055*	
C5	0.4472 (3)	0.4772 (2)	0.92296 (13)	0.0359 (5)	
H5	0.4244	0.4598	0.9651	0.043*	
C6	0.1226 (2)	0.61015 (17)	0.87053 (13)	0.0344 (4)	
C7	0.1880 (3)	0.6567 (2)	0.81897 (13)	0.0397 (6)	
H7	0.1663	0.6402	0.7766	0.048*	
C8	0.2910 (3)	0.7300 (2)	0.83914 (18)	0.0520 (8)	
H6	0.3520	0.7734	0.8132	0.062*	
C9	0.2912 (3)	0.7293 (2)	0.90302 (17)	0.0521 (8)	
H9	0.3516	0.7725	0.9293	0.062*	
C10	0.1877 (3)	0.6548 (2)	0.92278 (13)	0.0421 (6)	
H10	0.1647	0.6378	0.9650	0.051*	
C11	0.2823 (2)	0.34326 (16)	0.87073 (14)	0.0384 (5)	
C12	0.2072 (4)	0.3174 (3)	0.81283 (16)	0.0613 (9)	
H12A	0.2265	0.2466	0.8023	0.092*	
H12B	0.2418	0.3619	0.7812	0.092*	
H12C	0.1051	0.3270	0.8178	0.092*	
C13	0.0107 (2)	0.52915 (16)	0.86991 (14)	0.0354 (4)	
C14	−0.0273 (4)	0.4756 (3)	0.92816 (15)	0.0536 (8)	
H14A	−0.0365	0.4027	0.9210	0.080*	
H14B	−0.1171	0.5024	0.9432	0.080*	
H14C	0.0474	0.4876	0.9575	0.080*	
Fe2	0.39440 (3)	0.41401 (2)	0.617696 (15)	0.03361 (8)	
N5	0.7745 (2)	0.4900 (2)	0.68237 (11)	0.0429 (5)	
N6	0.8748 (3)	0.5680 (2)	0.68788 (14)	0.0566 (7)	
H6A	0.9232	0.5624	0.7231	0.068*	
H6B	0.9449	0.5782	0.6603	0.068*	
N7	0.4742 (3)	0.6966 (2)	0.55835 (14)	0.0597 (7)	
N8	0.5659 (4)	0.7824 (3)	0.5554 (2)	0.0937 (13)	
H8A	0.6512	0.7784	0.5747	0.112*	
H8B	0.6117	0.7617	0.5215	0.112*	
C15	0.6146 (2)	0.38990 (18)	0.62404 (13)	0.0355 (5)	
C16	0.5409 (3)	0.3428 (2)	0.67357 (13)	0.0389 (5)	
H16	0.5562	0.3588	0.7165	0.047*	
C17	0.4408 (4)	0.2695 (2)	0.65045 (16)	0.0487 (7)	
H17	0.3761	0.2259	0.6746	0.058*	
C18	0.4501 (4)	0.2712 (3)	0.58659 (16)	0.0528 (8)	
H18	0.3935	0.2286	0.5586	0.063*	
C19	0.5564 (3)	0.3450 (2)	0.56992 (13)	0.0448 (6)	
H19	0.5852	0.3623	0.5284	0.054*	
C20	0.3500 (2)	0.5705 (2)	0.61202 (14)	0.0377 (6)	
C21	0.2817 (3)	0.5239 (3)	0.66391 (14)	0.0484 (7)	
H21	0.2983	0.5437	0.7062	0.058*	
C22	0.1879 (3)	0.4450 (3)	0.64422 (18)	0.0571 (8)	
H22	0.1276	0.4013	0.6702	0.069*	
C23	0.1963 (3)	0.4401 (3)	0.58071 (16)	0.0516 (7)	
H23	0.1428	0.3920	0.5547	0.062*	
C24	0.2954 (3)	0.5164 (2)	0.56057 (14)	0.0414 (6)	
H24	0.3225	0.5294	0.5183	0.050*	
C25	0.7274 (2)	0.4692 (2)	0.62853 (12)	0.0378 (5)	
C26	0.7826 (4)	0.5208 (3)	0.57320 (15)	0.0534 (8)	
H26A	0.8859	0.5155	0.5721	0.080*	
H26B	0.7552	0.5925	0.5737	0.080*	
H26C	0.7424	0.4880	0.5380	0.080*	
C27	0.4541 (3)	0.6555 (2)	0.61029 (14)	0.0459 (6)	
C28	0.5251 (4)	0.6872 (3)	0.66867 (18)	0.0707 (11)	
H28A	0.6108	0.7263	0.6600	0.106*	
H28B	0.5504	0.6266	0.6915	0.106*	
H28C	0.4596	0.7290	0.6919	0.106*	

### Atomic displacement parameters (Å^2^)

**Table:**

	*U*^11^	*U*^22^	*U*^33^	*U*^12^	*U*^13^	*U*^23^
Fe1	0.02844 (15)	0.02768 (14)	0.04106 (17)	0.00246 (11)	0.00040 (14)	−0.00025 (17)
N1	0.0393 (11)	0.0360 (11)	0.0674 (15)	−0.0054 (9)	−0.0021 (11)	0.0015 (11)
N2	0.0499 (14)	0.0445 (14)	0.097 (2)	−0.0114 (11)	−0.0001 (14)	0.0051 (14)
N3	0.0365 (11)	0.0384 (11)	0.0504 (12)	0.0030 (9)	−0.0059 (9)	−0.0025 (10)
N4	0.0412 (12)	0.0508 (14)	0.0660 (16)	−0.0094 (10)	−0.0077 (11)	−0.0048 (12)
C1	0.0322 (11)	0.0333 (11)	0.0398 (12)	0.0096 (7)	0.0016 (13)	−0.0027 (11)
C2	0.0478 (15)	0.0489 (17)	0.0395 (13)	0.0177 (13)	0.0081 (12)	0.0014 (12)
C3	0.0342 (13)	0.0475 (17)	0.068 (2)	0.0055 (13)	0.0156 (13)	0.0129 (15)
C4	0.0313 (12)	0.0405 (14)	0.0667 (19)	0.0007 (11)	−0.0069 (13)	0.0046 (13)
C5	0.0355 (12)	0.0323 (12)	0.0399 (13)	0.0030 (10)	−0.0034 (10)	0.0019 (10)
C6	0.0293 (9)	0.0324 (10)	0.0415 (12)	0.0068 (7)	−0.0003 (12)	−0.0012 (11)
C7	0.0369 (13)	0.0358 (13)	0.0465 (14)	0.0081 (11)	−0.0014 (10)	0.0053 (11)
C8	0.0413 (16)	0.0286 (13)	0.086 (2)	−0.0005 (12)	0.0000 (15)	0.0107 (15)
C9	0.0460 (17)	0.0325 (14)	0.078 (2)	0.0036 (13)	−0.0045 (16)	−0.0140 (15)
C10	0.0382 (13)	0.0437 (14)	0.0446 (14)	0.0076 (11)	−0.0024 (11)	−0.0113 (11)
C11	0.0365 (10)	0.0282 (9)	0.0505 (12)	0.0065 (8)	−0.0044 (12)	−0.0049 (12)
C12	0.073 (2)	0.0441 (16)	0.0671 (19)	0.0066 (15)	−0.0256 (17)	−0.0143 (14)
C13	0.0272 (9)	0.0361 (11)	0.0429 (11)	0.0053 (8)	0.0025 (11)	−0.0009 (12)
C14	0.0494 (16)	0.064 (2)	0.0471 (16)	−0.0102 (15)	0.0060 (13)	0.0088 (15)
Fe2	0.02893 (15)	0.03346 (16)	0.03846 (16)	0.00323 (11)	−0.00165 (14)	0.00044 (17)
N5	0.0377 (10)	0.0441 (12)	0.0469 (12)	0.0003 (9)	−0.0073 (9)	−0.0050 (10)
N6	0.0481 (13)	0.0602 (16)	0.0615 (16)	−0.0142 (12)	−0.0100 (12)	−0.0059 (13)
N7	0.0462 (13)	0.0500 (15)	0.0828 (19)	−0.0088 (11)	−0.0112 (13)	0.0192 (14)
N8	0.0643 (19)	0.060 (2)	0.156 (4)	−0.0189 (17)	−0.021 (2)	0.030 (2)
C15	0.0294 (9)	0.0360 (10)	0.0411 (12)	0.0088 (9)	−0.0039 (10)	−0.0062 (11)
C16	0.0398 (13)	0.0327 (12)	0.0442 (13)	0.0082 (10)	−0.0013 (10)	0.0039 (10)
C17	0.0455 (16)	0.0312 (12)	0.070 (2)	0.0040 (12)	−0.0037 (14)	0.0037 (13)
C18	0.0478 (17)	0.0392 (15)	0.071 (2)	0.0039 (14)	−0.0113 (15)	−0.0182 (15)
C19	0.0422 (14)	0.0487 (15)	0.0436 (14)	0.0091 (12)	−0.0010 (11)	−0.0120 (12)
C20	0.0332 (12)	0.0370 (12)	0.0427 (14)	0.0122 (8)	−0.0009 (11)	0.0019 (12)
C21	0.0515 (16)	0.0501 (17)	0.0436 (15)	0.0243 (13)	0.0083 (12)	0.0032 (12)
C22	0.0354 (13)	0.0590 (19)	0.077 (2)	0.0086 (14)	0.0124 (14)	0.0185 (17)
C23	0.0310 (13)	0.0541 (17)	0.070 (2)	−0.0031 (12)	−0.0100 (13)	0.0107 (15)
C24	0.0342 (12)	0.0465 (16)	0.0435 (14)	0.0023 (11)	−0.0069 (11)	0.0051 (12)
C25	0.0303 (10)	0.0422 (12)	0.0410 (14)	0.0077 (9)	−0.0012 (10)	−0.0013 (11)
C26	0.0465 (15)	0.067 (2)	0.0473 (15)	−0.0066 (14)	0.0024 (12)	0.0045 (15)
C27	0.0417 (12)	0.0357 (12)	0.0603 (17)	0.0104 (10)	−0.0149 (13)	−0.0058 (13)
C28	0.080 (2)	0.0457 (17)	0.087 (3)	0.0142 (17)	−0.032 (2)	−0.0236 (17)

### Geometric parameters (Å, º)

**Table:**

Fe1—C10	2.035 (3)	Fe2—C18	2.036 (3)
Fe1—C9	2.035 (3)	Fe2—C21	2.037 (3)
Fe1—C3	2.036 (3)	Fe2—C23	2.037 (3)
Fe1—C2	2.042 (3)	Fe2—C19	2.040 (3)
Fe1—C8	2.046 (3)	Fe2—C22	2.040 (3)
Fe1—C4	2.049 (3)	Fe2—C24	2.045 (3)
Fe1—C7	2.053 (3)	Fe2—C17	2.048 (3)
Fe1—C5	2.055 (3)	Fe2—C16	2.052 (3)
Fe1—C6	2.0624 (19)	Fe2—C20	2.067 (3)
Fe1—C1	2.065 (2)	Fe2—C15	2.069 (2)
N1—C11	1.276 (4)	N5—C25	1.294 (4)
N1—N2	1.410 (3)	N5—N6	1.377 (3)
N2—H2A	0.8996	N6—H6A	0.9003
N2—H2B	0.9002	N6—H6B	0.8993
N3—C13	1.276 (4)	N7—C27	1.277 (4)
N3—N4	1.394 (3)	N7—N8	1.398 (4)
N4—H4A	0.9002	N8—H8A	0.8998
N4—H4B	0.9004	N8—H8B	0.9005
C1—C5	1.424 (4)	C15—C16	1.425 (4)
C1—C2	1.430 (4)	C15—C19	1.433 (4)
C1—C11	1.472 (3)	C15—C25	1.466 (3)
C2—C3	1.416 (5)	C16—C17	1.421 (4)
C2—H2	0.9800	C16—H16	0.9800
C3—C4	1.412 (4)	C17—C18	1.413 (4)
C3—H3	0.9800	C17—H17	0.9800
C4—C5	1.426 (4)	C18—C19	1.420 (5)
C4—H4	0.9800	C18—H18	0.9800
C5—H5	0.9800	C19—H19	0.9800
C6—C7	1.423 (4)	C20—C24	1.426 (4)
C6—C10	1.424 (4)	C20—C21	1.440 (4)
C6—C13	1.473 (3)	C20—C27	1.463 (4)
C7—C8	1.417 (4)	C21—C22	1.409 (5)
C7—H7	0.9800	C21—H21	0.9800
C8—C9	1.410 (4)	C22—C23	1.405 (5)
C8—H6	0.9800	C22—H22	0.9800
C9—C10	1.427 (5)	C23—C24	1.419 (4)
C9—H9	0.9800	C23—H23	0.9800
C10—H10	0.9800	C24—H24	0.9800
C11—C12	1.493 (4)	C25—C26	1.483 (4)
C12—H12A	0.9600	C26—H26A	0.9600
C12—H12B	0.9600	C26—H26B	0.9600
C12—H12C	0.9600	C26—H26C	0.9600
C13—C14	1.502 (4)	C27—C28	1.504 (4)
C14—H14A	0.9600	C28—H28A	0.9600
C14—H14B	0.9600	C28—H28B	0.9600
C14—H14C	0.9600	C28—H28C	0.9600
			
C10—Fe1—C9	41.05 (13)	C18—Fe2—C21	158.63 (15)
C10—Fe1—C3	157.39 (13)	C18—Fe2—C23	104.11 (14)
C9—Fe1—C3	120.43 (14)	C21—Fe2—C23	67.87 (14)
C10—Fe1—C2	160.90 (13)	C18—Fe2—C19	40.77 (14)
C9—Fe1—C2	156.84 (14)	C21—Fe2—C19	160.22 (14)
C3—Fe1—C2	40.63 (14)	C23—Fe2—C19	121.90 (13)
C10—Fe1—C8	68.45 (14)	C18—Fe2—C22	120.85 (15)
C9—Fe1—C8	40.42 (12)	C21—Fe2—C22	40.42 (15)
C3—Fe1—C8	105.66 (13)	C23—Fe2—C22	40.32 (14)
C2—Fe1—C8	121.94 (14)	C19—Fe2—C22	157.55 (15)
C10—Fe1—C4	122.37 (12)	C18—Fe2—C24	119.51 (14)
C9—Fe1—C4	105.66 (13)	C21—Fe2—C24	68.13 (12)
C3—Fe1—C4	40.44 (12)	C23—Fe2—C24	40.68 (12)
C2—Fe1—C4	68.35 (13)	C19—Fe2—C24	107.11 (13)
C8—Fe1—C4	120.73 (13)	C22—Fe2—C24	68.24 (13)
C10—Fe1—C7	68.20 (9)	C18—Fe2—C17	40.47 (12)
C9—Fe1—C7	68.08 (13)	C21—Fe2—C17	124.51 (13)
C3—Fe1—C7	122.33 (13)	C23—Fe2—C17	118.79 (13)
C2—Fe1—C7	108.31 (12)	C19—Fe2—C17	68.27 (14)
C8—Fe1—C7	40.44 (12)	C22—Fe2—C17	105.93 (14)
C4—Fe1—C7	157.23 (12)	C24—Fe2—C17	154.40 (13)
C10—Fe1—C5	108.69 (12)	C18—Fe2—C16	68.19 (12)
C9—Fe1—C5	122.80 (13)	C21—Fe2—C16	110.53 (12)
C3—Fe1—C5	68.01 (12)	C23—Fe2—C16	155.69 (12)
C2—Fe1—C5	68.10 (10)	C19—Fe2—C16	68.16 (10)
C8—Fe1—C5	157.67 (13)	C22—Fe2—C16	122.40 (13)
C4—Fe1—C5	40.66 (11)	C24—Fe2—C16	163.21 (11)
C7—Fe1—C5	160.95 (11)	C17—Fe2—C16	40.56 (12)
C10—Fe1—C6	40.67 (10)	C18—Fe2—C20	156.46 (14)
C9—Fe1—C6	68.55 (11)	C21—Fe2—C20	41.08 (12)
C3—Fe1—C6	159.37 (13)	C23—Fe2—C20	68.55 (12)
C2—Fe1—C6	124.55 (12)	C19—Fe2—C20	122.86 (12)
C8—Fe1—C6	68.26 (11)	C22—Fe2—C20	68.83 (13)
C4—Fe1—C6	159.72 (12)	C24—Fe2—C20	40.57 (12)
C7—Fe1—C6	40.46 (11)	C17—Fe2—C20	162.74 (13)
C5—Fe1—C6	124.97 (11)	C16—Fe2—C20	127.34 (11)
C10—Fe1—C1	124.59 (12)	C18—Fe2—C15	68.66 (12)
C9—Fe1—C1	159.87 (14)	C21—Fe2—C15	125.21 (12)
C3—Fe1—C1	68.40 (11)	C23—Fe2—C15	160.19 (13)
C2—Fe1—C1	40.76 (12)	C19—Fe2—C15	40.83 (11)
C8—Fe1—C1	159.23 (14)	C22—Fe2—C15	159.21 (13)
C4—Fe1—C1	68.48 (11)	C24—Fe2—C15	125.55 (11)
C7—Fe1—C1	124.67 (11)	C17—Fe2—C15	68.41 (12)
C5—Fe1—C1	40.42 (11)	C16—Fe2—C15	40.48 (11)
C6—Fe1—C1	109.90 (9)	C20—Fe2—C15	110.34 (9)
C11—N1—N2	117.8 (3)	C25—N5—N6	117.5 (3)
N1—N2—H2A	83.2	N5—N6—H6A	110.7
N1—N2—H2B	99.3	N5—N6—H6B	122.4
H2A—N2—H2B	120.7	H6A—N6—H6B	103.7
C13—N3—N4	118.1 (3)	C27—N7—N8	117.4 (3)
N3—N4—H4A	104.7	N7—N8—H8A	117.7
N3—N4—H4B	117.7	N7—N8—H8B	95.2
H4A—N4—H4B	114.2	H8A—N8—H8B	87.9
C5—C1—C2	107.0 (2)	C16—C15—C19	106.6 (2)
C5—C1—C11	126.0 (3)	C16—C15—C25	126.0 (2)
C2—C1—C11	127.0 (3)	C19—C15—C25	127.4 (3)
C5—C1—Fe1	69.42 (14)	C16—C15—Fe2	69.11 (14)
C2—C1—Fe1	68.76 (15)	C19—C15—Fe2	68.49 (14)
C11—C1—Fe1	127.97 (14)	C25—C15—Fe2	127.00 (16)
C3—C2—C1	108.2 (3)	C17—C16—C15	108.8 (3)
C3—C2—Fe1	69.44 (18)	C17—C16—Fe2	69.59 (16)
C1—C2—Fe1	70.48 (15)	C15—C16—Fe2	70.41 (14)
C3—C2—H2	125.9	C17—C16—H16	125.6
C1—C2—H2	125.9	C15—C16—H16	125.6
Fe1—C2—H2	125.9	Fe2—C16—H16	125.6
C4—C3—C2	108.7 (3)	C18—C17—C16	107.9 (3)
C4—C3—Fe1	70.27 (19)	C18—C17—Fe2	69.3 (2)
C2—C3—Fe1	69.93 (17)	C16—C17—Fe2	69.86 (16)
C4—C3—H3	125.6	C18—C17—H17	126.0
C2—C3—H3	125.6	C16—C17—H17	126.0
Fe1—C3—H3	125.6	Fe2—C17—H17	126.0
C3—C4—C5	107.5 (3)	C17—C18—C19	108.2 (3)
C3—C4—Fe1	69.29 (19)	C17—C18—Fe2	70.2 (2)
C5—C4—Fe1	69.92 (15)	C19—C18—Fe2	69.76 (17)
C3—C4—H4	126.3	C17—C18—H18	125.9
C5—C4—H4	126.3	C19—C18—H18	125.9
Fe1—C4—H4	126.3	Fe2—C18—H18	125.9
C1—C5—C4	108.7 (3)	C18—C19—C15	108.5 (3)
C1—C5—Fe1	70.15 (14)	C18—C19—Fe2	69.47 (18)
C4—C5—Fe1	69.42 (16)	C15—C19—Fe2	70.68 (14)
C1—C5—H5	125.7	C18—C19—H19	125.8
C4—C5—H5	125.7	C15—C19—H19	125.8
Fe1—C5—H5	125.7	Fe2—C19—H19	125.8
C7—C6—C10	107.2 (2)	C24—C20—C21	105.8 (2)
C7—C6—C13	126.4 (3)	C24—C20—C27	125.5 (3)
C10—C6—C13	126.4 (3)	C21—C20—C27	128.6 (3)
C7—C6—Fe1	69.40 (13)	C24—C20—Fe2	68.89 (16)
C10—C6—Fe1	68.63 (13)	C21—C20—Fe2	68.31 (16)
C13—C6—Fe1	126.05 (15)	C27—C20—Fe2	127.28 (16)
C8—C7—C6	108.6 (3)	C22—C21—C20	109.2 (3)
C8—C7—Fe1	69.54 (17)	C22—C21—Fe2	69.94 (18)
C6—C7—Fe1	70.14 (13)	C20—C21—Fe2	70.61 (15)
C8—C7—H7	125.7	C22—C21—H21	125.4
C6—C7—H7	125.7	C20—C21—H21	125.4
Fe1—C7—H7	125.7	Fe2—C21—H21	125.4
C9—C8—C7	108.1 (3)	C23—C22—C21	107.8 (3)
C9—C8—Fe1	69.4 (2)	C23—C22—Fe2	69.72 (19)
C7—C8—Fe1	70.02 (16)	C21—C22—Fe2	69.64 (17)
C9—C8—H6	126.0	C23—C22—H22	126.1
C7—C8—H6	126.0	C21—C22—H22	126.1
Fe1—C8—H6	126.0	Fe2—C22—H22	126.1
C8—C9—C10	108.0 (3)	C22—C23—C24	108.5 (3)
C8—C9—Fe1	70.2 (2)	C22—C23—Fe2	69.96 (19)
C10—C9—Fe1	69.47 (16)	C24—C23—Fe2	69.97 (16)
C8—C9—H9	126.0	C22—C23—H23	125.8
C10—C9—H9	126.0	C24—C23—H23	125.8
Fe1—C9—H9	126.0	Fe2—C23—H23	125.8
C6—C10—C9	108.1 (3)	C23—C24—C20	108.7 (3)
C6—C10—Fe1	70.71 (13)	C23—C24—Fe2	69.35 (17)
C9—C10—Fe1	69.48 (17)	C20—C24—Fe2	70.54 (15)
C6—C10—H10	126.0	C23—C24—H24	125.7
C9—C10—H10	126.0	C20—C24—H24	125.7
Fe1—C10—H10	126.0	Fe2—C24—H24	125.7
N1—C11—C1	115.7 (3)	N5—C25—C15	116.6 (3)
N1—C11—C12	126.0 (2)	N5—C25—C26	123.1 (3)
C1—C11—C12	118.3 (3)	C15—C25—C26	120.3 (3)
C11—C12—H12A	109.5	C25—C26—H26A	109.5
C11—C12—H12B	109.5	C25—C26—H26B	109.5
H12A—C12—H12B	109.5	H26A—C26—H26B	109.5
C11—C12—H12C	109.5	C25—C26—H26C	109.5
H12A—C12—H12C	109.5	H26A—C26—H26C	109.5
H12B—C12—H12C	109.5	H26B—C26—H26C	109.5
N3—C13—C6	116.5 (3)	N7—C27—C20	115.6 (3)
N3—C13—C14	124.5 (2)	N7—C27—C28	126.3 (3)
C6—C13—C14	119.0 (3)	C20—C27—C28	118.1 (3)
C13—C14—H14A	109.5	C27—C28—H28A	109.5
C13—C14—H14B	109.5	C27—C28—H28B	109.5
H14A—C14—H14B	109.5	H28A—C28—H28B	109.5
C13—C14—H14C	109.5	C27—C28—H28C	109.5
H14A—C14—H14C	109.5	H28A—C28—H28C	109.5
H14B—C14—H14C	109.5	H28B—C28—H28C	109.5
			
C10—Fe1—C1—C5	−77.91 (18)	C18—Fe2—C15—C16	−80.99 (18)
C9—Fe1—C1—C5	−39.2 (4)	C21—Fe2—C15—C16	80.46 (19)
C3—Fe1—C1—C5	81.00 (19)	C23—Fe2—C15—C16	−152.8 (3)
C2—Fe1—C1—C5	118.7 (2)	C19—Fe2—C15—C16	−118.7 (2)
C8—Fe1—C1—C5	158.0 (3)	C22—Fe2—C15—C16	40.5 (4)
C4—Fe1—C1—C5	37.37 (17)	C24—Fe2—C15—C16	167.25 (17)
C7—Fe1—C1—C5	−163.81 (16)	C17—Fe2—C15—C16	−37.35 (17)
C6—Fe1—C1—C5	−121.00 (16)	C20—Fe2—C15—C16	124.21 (16)
C10—Fe1—C1—C2	163.35 (18)	C18—Fe2—C15—C19	37.68 (19)
C9—Fe1—C1—C2	−157.9 (3)	C21—Fe2—C15—C19	−160.88 (18)
C3—Fe1—C1—C2	−37.74 (19)	C23—Fe2—C15—C19	−34.1 (4)
C8—Fe1—C1—C2	39.3 (4)	C22—Fe2—C15—C19	159.1 (3)
C4—Fe1—C1—C2	−81.4 (2)	C24—Fe2—C15—C19	−74.1 (2)
C7—Fe1—C1—C2	77.4 (2)	C17—Fe2—C15—C19	81.3 (2)
C5—Fe1—C1—C2	−118.7 (2)	C16—Fe2—C15—C19	118.7 (2)
C6—Fe1—C1—C2	120.26 (18)	C20—Fe2—C15—C19	−117.13 (18)
C10—Fe1—C1—C11	42.4 (3)	C18—Fe2—C15—C25	159.0 (3)
C9—Fe1—C1—C11	81.1 (4)	C21—Fe2—C15—C25	−39.5 (3)
C3—Fe1—C1—C11	−158.7 (3)	C23—Fe2—C15—C25	87.2 (4)
C2—Fe1—C1—C11	−121.0 (4)	C19—Fe2—C15—C25	121.3 (3)
C8—Fe1—C1—C11	−81.7 (4)	C22—Fe2—C15—C25	−79.5 (4)
C4—Fe1—C1—C11	157.6 (3)	C24—Fe2—C15—C25	47.2 (3)
C7—Fe1—C1—C11	−43.5 (3)	C17—Fe2—C15—C25	−157.4 (3)
C5—Fe1—C1—C11	120.3 (3)	C16—Fe2—C15—C25	−120.0 (3)
C6—Fe1—C1—C11	−0.7 (3)	C20—Fe2—C15—C25	4.2 (3)
C5—C1—C2—C3	0.3 (3)	C19—C15—C16—C17	0.8 (3)
C11—C1—C2—C3	−178.3 (2)	C25—C15—C16—C17	−179.6 (2)
Fe1—C1—C2—C3	59.4 (2)	Fe2—C15—C16—C17	59.19 (19)
C5—C1—C2—Fe1	−59.13 (15)	C19—C15—C16—Fe2	−58.43 (15)
C11—C1—C2—Fe1	122.2 (2)	C25—C15—C16—Fe2	121.3 (2)
C10—Fe1—C2—C3	−165.2 (3)	C18—Fe2—C16—C17	−37.6 (2)
C9—Fe1—C2—C3	41.7 (4)	C21—Fe2—C16—C17	119.5 (2)
C8—Fe1—C2—C3	76.2 (2)	C23—Fe2—C16—C17	38.1 (4)
C4—Fe1—C2—C3	−37.36 (18)	C19—Fe2—C16—C17	−81.6 (2)
C7—Fe1—C2—C3	118.65 (19)	C22—Fe2—C16—C17	76.0 (2)
C5—Fe1—C2—C3	−81.3 (2)	C24—Fe2—C16—C17	−158.3 (4)
C6—Fe1—C2—C3	160.46 (18)	C20—Fe2—C16—C17	162.92 (19)
C1—Fe1—C2—C3	−119.1 (3)	C15—Fe2—C16—C17	−119.8 (2)
C10—Fe1—C2—C1	−46.1 (4)	C18—Fe2—C16—C15	82.25 (18)
C9—Fe1—C2—C1	160.8 (3)	C21—Fe2—C16—C15	−120.64 (17)
C3—Fe1—C2—C1	119.1 (3)	C23—Fe2—C16—C15	157.9 (3)
C8—Fe1—C2—C1	−164.67 (16)	C19—Fe2—C16—C15	38.17 (15)
C4—Fe1—C2—C1	81.73 (18)	C22—Fe2—C16—C15	−164.17 (17)
C7—Fe1—C2—C1	−122.26 (16)	C24—Fe2—C16—C15	−38.5 (5)
C5—Fe1—C2—C1	37.79 (15)	C17—Fe2—C16—C15	119.8 (2)
C6—Fe1—C2—C1	−80.44 (19)	C20—Fe2—C16—C15	−77.26 (18)
C1—C2—C3—C4	−0.3 (4)	C15—C16—C17—C18	−0.7 (3)
Fe1—C2—C3—C4	59.8 (2)	Fe2—C16—C17—C18	59.0 (2)
C1—C2—C3—Fe1	−60.09 (18)	C15—C16—C17—Fe2	−59.70 (17)
C10—Fe1—C3—C4	47.9 (4)	C21—Fe2—C17—C18	159.3 (2)
C9—Fe1—C3—C4	78.1 (2)	C23—Fe2—C17—C18	77.6 (3)
C2—Fe1—C3—C4	−119.6 (3)	C19—Fe2—C17—C18	−37.9 (2)
C8—Fe1—C3—C4	119.3 (2)	C22—Fe2—C17—C18	119.1 (2)
C7—Fe1—C3—C4	160.00 (19)	C24—Fe2—C17—C18	46.4 (4)
C5—Fe1—C3—C4	−38.06 (19)	C16—Fe2—C17—C18	−119.3 (3)
C6—Fe1—C3—C4	−171.0 (2)	C20—Fe2—C17—C18	−171.2 (3)
C1—Fe1—C3—C4	−81.7 (2)	C15—Fe2—C17—C18	−82.0 (2)
C10—Fe1—C3—C2	167.5 (3)	C18—Fe2—C17—C16	119.3 (3)
C9—Fe1—C3—C2	−162.33 (19)	C21—Fe2—C17—C16	−81.4 (2)
C8—Fe1—C3—C2	−121.1 (2)	C23—Fe2—C17—C16	−163.17 (18)
C4—Fe1—C3—C2	119.6 (3)	C19—Fe2—C17—C16	81.37 (18)
C7—Fe1—C3—C2	−80.4 (2)	C22—Fe2—C17—C16	−121.6 (2)
C5—Fe1—C3—C2	81.54 (17)	C24—Fe2—C17—C16	165.7 (3)
C6—Fe1—C3—C2	−51.4 (4)	C20—Fe2—C17—C16	−51.9 (5)
C1—Fe1—C3—C2	37.85 (18)	C15—Fe2—C17—C16	37.28 (17)
C2—C3—C4—C5	0.2 (4)	C16—C17—C18—C19	0.3 (4)
Fe1—C3—C4—C5	59.7 (2)	Fe2—C17—C18—C19	59.7 (2)
C2—C3—C4—Fe1	−59.6 (2)	C16—C17—C18—Fe2	−59.4 (2)
C10—Fe1—C4—C3	−160.3 (2)	C21—Fe2—C18—C17	−53.1 (5)
C9—Fe1—C4—C3	−118.8 (2)	C23—Fe2—C18—C17	−118.1 (2)
C2—Fe1—C4—C3	37.5 (2)	C19—Fe2—C18—C17	119.1 (3)
C8—Fe1—C4—C3	−77.7 (3)	C22—Fe2—C18—C17	−78.0 (3)
C7—Fe1—C4—C3	−48.3 (4)	C24—Fe2—C18—C17	−158.9 (2)
C5—Fe1—C4—C3	118.7 (3)	C16—Fe2—C18—C17	37.7 (2)
C6—Fe1—C4—C3	170.9 (3)	C20—Fe2—C18—C17	173.5 (2)
C1—Fe1—C4—C3	81.5 (2)	C15—Fe2—C18—C17	81.3 (2)
C10—Fe1—C4—C5	81.0 (2)	C21—Fe2—C18—C19	−172.2 (3)
C9—Fe1—C4—C5	122.49 (19)	C23—Fe2—C18—C19	122.88 (19)
C3—Fe1—C4—C5	−118.7 (3)	C22—Fe2—C18—C19	162.92 (18)
C2—Fe1—C4—C5	−81.15 (18)	C24—Fe2—C18—C19	82.0 (2)
C8—Fe1—C4—C5	163.63 (19)	C17—Fe2—C18—C19	−119.1 (3)
C7—Fe1—C4—C5	−167.0 (3)	C16—Fe2—C18—C19	−81.41 (18)
C6—Fe1—C4—C5	52.2 (4)	C20—Fe2—C18—C19	54.4 (4)
C1—Fe1—C4—C5	−37.17 (17)	C15—Fe2—C18—C19	−37.73 (17)
C2—C1—C5—C4	−0.2 (3)	C17—C18—C19—C15	0.2 (4)
C11—C1—C5—C4	178.4 (2)	Fe2—C18—C19—C15	60.16 (18)
Fe1—C1—C5—C4	−58.92 (18)	C17—C18—C19—Fe2	−60.0 (3)
C2—C1—C5—Fe1	58.72 (16)	C16—C15—C19—C18	−0.6 (3)
C11—C1—C5—Fe1	−122.6 (2)	C25—C15—C19—C18	179.7 (2)
C3—C4—C5—C1	0.0 (3)	Fe2—C15—C19—C18	−59.4 (2)
Fe1—C4—C5—C1	59.37 (18)	C16—C15—C19—Fe2	58.82 (15)
C3—C4—C5—Fe1	−59.3 (2)	C25—C15—C19—Fe2	−120.9 (2)
C10—Fe1—C5—C1	121.82 (15)	C21—Fe2—C19—C18	171.6 (3)
C9—Fe1—C5—C1	165.00 (16)	C23—Fe2—C19—C18	−73.6 (2)
C3—Fe1—C5—C1	−82.07 (19)	C22—Fe2—C19—C18	−41.3 (4)
C2—Fe1—C5—C1	−38.10 (15)	C24—Fe2—C19—C18	−115.6 (2)
C8—Fe1—C5—C1	−159.5 (3)	C17—Fe2—C19—C18	37.65 (19)
C4—Fe1—C5—C1	−119.9 (2)	C16—Fe2—C19—C18	81.5 (2)
C7—Fe1—C5—C1	44.6 (4)	C20—Fe2—C19—C18	−157.25 (18)
C6—Fe1—C5—C1	79.60 (18)	C15—Fe2—C19—C18	119.3 (3)
C10—Fe1—C5—C4	−118.26 (19)	C18—Fe2—C19—C15	−119.3 (3)
C9—Fe1—C5—C4	−75.1 (2)	C21—Fe2—C19—C15	52.3 (4)
C3—Fe1—C5—C4	37.85 (19)	C23—Fe2—C19—C15	167.06 (16)
C2—Fe1—C5—C4	81.8 (2)	C22—Fe2—C19—C15	−160.7 (3)
C8—Fe1—C5—C4	−39.6 (4)	C24—Fe2—C19—C15	125.05 (17)
C7—Fe1—C5—C4	164.5 (3)	C17—Fe2—C19—C15	−81.68 (18)
C6—Fe1—C5—C4	−160.48 (18)	C16—Fe2—C19—C15	−37.85 (15)
C1—Fe1—C5—C4	119.9 (2)	C20—Fe2—C19—C15	83.42 (19)
C10—Fe1—C6—C7	119.1 (2)	C18—Fe2—C20—C24	38.3 (3)
C9—Fe1—C6—C7	80.95 (18)	C21—Fe2—C20—C24	−117.9 (2)
C3—Fe1—C6—C7	−39.1 (4)	C23—Fe2—C20—C24	−37.44 (18)
C2—Fe1—C6—C7	−77.31 (18)	C19—Fe2—C20—C24	77.53 (19)
C8—Fe1—C6—C7	37.32 (18)	C22—Fe2—C20—C24	−80.86 (19)
C4—Fe1—C6—C7	157.9 (3)	C17—Fe2—C20—C24	−156.1 (4)
C5—Fe1—C6—C7	−163.23 (16)	C16—Fe2—C20—C24	163.84 (17)
C1—Fe1—C6—C7	−120.52 (17)	C15—Fe2—C20—C24	121.37 (17)
C9—Fe1—C6—C10	−38.18 (19)	C18—Fe2—C20—C21	156.2 (3)
C3—Fe1—C6—C10	−158.3 (3)	C23—Fe2—C20—C21	80.5 (2)
C2—Fe1—C6—C10	163.56 (18)	C19—Fe2—C20—C21	−164.55 (18)
C8—Fe1—C6—C10	−81.8 (2)	C22—Fe2—C20—C21	37.1 (2)
C4—Fe1—C6—C10	38.7 (4)	C24—Fe2—C20—C21	117.9 (2)
C7—Fe1—C6—C10	−119.1 (2)	C17—Fe2—C20—C21	−38.2 (4)
C5—Fe1—C6—C10	77.64 (19)	C16—Fe2—C20—C21	−78.2 (2)
C1—Fe1—C6—C10	120.35 (18)	C15—Fe2—C20—C21	−120.71 (18)
C10—Fe1—C6—C13	−120.2 (3)	C18—Fe2—C20—C27	−81.0 (4)
C9—Fe1—C6—C13	−158.4 (3)	C21—Fe2—C20—C27	122.8 (4)
C3—Fe1—C6—C13	81.5 (4)	C23—Fe2—C20—C27	−156.7 (3)
C2—Fe1—C6—C13	43.3 (3)	C19—Fe2—C20—C27	−41.8 (3)
C8—Fe1—C6—C13	158.0 (3)	C22—Fe2—C20—C27	159.9 (3)
C4—Fe1—C6—C13	−81.5 (4)	C24—Fe2—C20—C27	−119.3 (4)
C7—Fe1—C6—C13	120.6 (3)	C17—Fe2—C20—C27	84.6 (5)
C5—Fe1—C6—C13	−42.6 (3)	C16—Fe2—C20—C27	44.6 (3)
C1—Fe1—C6—C13	0.1 (3)	C15—Fe2—C20—C27	2.1 (3)
C10—C6—C7—C8	−0.7 (3)	C24—C20—C21—C22	−0.6 (3)
C13—C6—C7—C8	−179.4 (2)	C27—C20—C21—C22	179.4 (2)
Fe1—C6—C7—C8	−59.12 (19)	Fe2—C20—C21—C22	−59.5 (2)
C10—C6—C7—Fe1	58.39 (15)	C24—C20—C21—Fe2	58.96 (17)
C13—C6—C7—Fe1	−120.3 (2)	C27—C20—C21—Fe2	−121.1 (2)
C10—Fe1—C7—C8	81.9 (2)	C18—Fe2—C21—C22	−33.9 (4)
C9—Fe1—C7—C8	37.5 (2)	C23—Fe2—C21—C22	37.6 (2)
C3—Fe1—C7—C8	−75.5 (2)	C19—Fe2—C21—C22	161.3 (3)
C2—Fe1—C7—C8	−118.1 (2)	C24—Fe2—C21—C22	81.7 (2)
C4—Fe1—C7—C8	−40.5 (4)	C17—Fe2—C21—C22	−72.9 (2)
C5—Fe1—C7—C8	166.1 (3)	C16—Fe2—C21—C22	−116.3 (2)
C6—Fe1—C7—C8	119.7 (3)	C20—Fe2—C21—C22	119.9 (3)
C1—Fe1—C7—C8	−160.25 (19)	C15—Fe2—C21—C22	−159.41 (19)
C10—Fe1—C7—C6	−37.81 (14)	C18—Fe2—C21—C20	−153.8 (3)
C9—Fe1—C7—C6	−82.21 (18)	C23—Fe2—C21—C20	−82.29 (18)
C3—Fe1—C7—C6	164.74 (16)	C19—Fe2—C21—C20	41.4 (4)
C2—Fe1—C7—C6	122.18 (17)	C22—Fe2—C21—C20	−119.9 (3)
C8—Fe1—C7—C6	−119.7 (3)	C24—Fe2—C21—C20	−38.26 (16)
C4—Fe1—C7—C6	−160.3 (3)	C17—Fe2—C21—C20	167.15 (17)
C5—Fe1—C7—C6	46.4 (4)	C16—Fe2—C21—C20	123.79 (16)
C1—Fe1—C7—C6	80.02 (18)	C15—Fe2—C21—C20	80.66 (19)
C6—C7—C8—C9	0.4 (3)	C20—C21—C22—C23	0.4 (3)
Fe1—C7—C8—C9	−59.1 (2)	Fe2—C21—C22—C23	−59.5 (2)
C6—C7—C8—Fe1	59.48 (17)	C20—C21—C22—Fe2	59.93 (18)
C10—Fe1—C8—C9	38.1 (2)	C18—Fe2—C22—C23	−74.6 (3)
C3—Fe1—C8—C9	−118.8 (2)	C21—Fe2—C22—C23	119.0 (3)
C2—Fe1—C8—C9	−159.9 (2)	C19—Fe2—C22—C23	−44.5 (4)
C4—Fe1—C8—C9	−77.7 (3)	C24—Fe2—C22—C23	37.7 (2)
C7—Fe1—C8—C9	119.4 (3)	C17—Fe2—C22—C23	−116.0 (2)
C5—Fe1—C8—C9	−48.8 (5)	C16—Fe2—C22—C23	−156.96 (19)
C6—Fe1—C8—C9	82.0 (2)	C20—Fe2—C22—C23	81.4 (2)
C1—Fe1—C8—C9	171.0 (2)	C15—Fe2—C22—C23	173.1 (3)
C10—Fe1—C8—C7	−81.24 (18)	C18—Fe2—C22—C21	166.33 (19)
C9—Fe1—C8—C7	−119.4 (3)	C23—Fe2—C22—C21	−119.0 (3)
C3—Fe1—C8—C7	121.8 (2)	C19—Fe2—C22—C21	−163.5 (3)
C2—Fe1—C8—C7	80.8 (2)	C24—Fe2—C22—C21	−81.37 (19)
C4—Fe1—C8—C7	162.98 (18)	C17—Fe2—C22—C21	125.0 (2)
C5—Fe1—C8—C7	−168.1 (3)	C16—Fe2—C22—C21	84.0 (2)
C6—Fe1—C8—C7	−37.34 (17)	C20—Fe2—C22—C21	−37.64 (18)
C1—Fe1—C8—C7	51.6 (4)	C15—Fe2—C22—C21	54.1 (4)
C7—C8—C9—C10	0.1 (4)	C21—C22—C23—C24	−0.2 (4)
Fe1—C8—C9—C10	−59.4 (2)	Fe2—C22—C23—C24	−59.6 (2)
C7—C8—C9—Fe1	59.5 (2)	C21—C22—C23—Fe2	59.4 (2)
C10—Fe1—C9—C8	−119.1 (3)	C18—Fe2—C23—C22	121.4 (2)
C3—Fe1—C9—C8	78.1 (3)	C21—Fe2—C23—C22	−37.7 (2)
C2—Fe1—C9—C8	47.9 (4)	C19—Fe2—C23—C22	161.6 (2)
C4—Fe1—C9—C8	119.3 (2)	C24—Fe2—C23—C22	−119.5 (3)
C7—Fe1—C9—C8	−37.5 (2)	C17—Fe2—C23—C22	80.6 (3)
C5—Fe1—C9—C8	160.1 (2)	C16—Fe2—C23—C22	53.4 (4)
C6—Fe1—C9—C8	−81.2 (2)	C20—Fe2—C23—C22	−82.1 (2)
C1—Fe1—C9—C8	−170.7 (2)	C15—Fe2—C23—C22	−172.8 (3)
C3—Fe1—C9—C10	−162.88 (17)	C18—Fe2—C23—C24	−119.1 (2)
C2—Fe1—C9—C10	167.0 (3)	C21—Fe2—C23—C24	81.7 (2)
C8—Fe1—C9—C10	119.1 (3)	C19—Fe2—C23—C24	−78.9 (2)
C4—Fe1—C9—C10	−121.65 (18)	C22—Fe2—C23—C24	119.5 (3)
C7—Fe1—C9—C10	81.52 (17)	C17—Fe2—C23—C24	−159.94 (19)
C5—Fe1—C9—C10	−80.8 (2)	C16—Fe2—C23—C24	172.8 (3)
C6—Fe1—C9—C10	37.83 (17)	C20—Fe2—C23—C24	37.34 (19)
C1—Fe1—C9—C10	−51.6 (4)	C15—Fe2—C23—C24	−53.3 (4)
C7—C6—C10—C9	0.8 (3)	C22—C23—C24—C20	−0.2 (4)
C13—C6—C10—C9	179.4 (2)	Fe2—C23—C24—C20	−59.78 (19)
Fe1—C6—C10—C9	59.67 (19)	C22—C23—C24—Fe2	59.6 (2)
C7—C6—C10—Fe1	−58.88 (15)	C21—C20—C24—C23	0.5 (3)
C13—C6—C10—Fe1	119.8 (2)	C27—C20—C24—C23	−179.5 (2)
C8—C9—C10—C6	−0.6 (4)	Fe2—C20—C24—C23	59.0 (2)
Fe1—C9—C10—C6	−60.44 (18)	C21—C20—C24—Fe2	−58.58 (16)
C8—C9—C10—Fe1	59.9 (2)	C27—C20—C24—Fe2	121.5 (2)
C9—Fe1—C10—C6	118.8 (3)	C18—Fe2—C24—C23	76.8 (2)
C3—Fe1—C10—C6	160.2 (3)	C21—Fe2—C24—C23	−81.0 (2)
C2—Fe1—C10—C6	−45.4 (4)	C19—Fe2—C24—C23	119.3 (2)
C8—Fe1—C10—C6	81.29 (18)	C22—Fe2—C24—C23	−37.3 (2)
C4—Fe1—C10—C6	−165.12 (15)	C17—Fe2—C24—C23	44.1 (4)
C7—Fe1—C10—C6	37.62 (14)	C16—Fe2—C24—C23	−169.8 (4)
C5—Fe1—C10—C6	−122.32 (16)	C20—Fe2—C24—C23	−119.8 (3)
C1—Fe1—C10—C6	−80.30 (19)	C15—Fe2—C24—C23	160.48 (19)
C3—Fe1—C10—C9	41.3 (4)	C18—Fe2—C24—C20	−163.47 (17)
C2—Fe1—C10—C9	−164.3 (3)	C21—Fe2—C24—C20	38.73 (16)
C8—Fe1—C10—C9	−37.54 (19)	C23—Fe2—C24—C20	119.8 (3)
C4—Fe1—C10—C9	76.0 (2)	C19—Fe2—C24—C20	−120.89 (17)
C7—Fe1—C10—C9	−81.2 (2)	C22—Fe2—C24—C20	82.4 (2)
C5—Fe1—C10—C9	118.8 (2)	C17—Fe2—C24—C20	163.8 (3)
C6—Fe1—C10—C9	−118.8 (3)	C16—Fe2—C24—C20	−50.0 (5)
C1—Fe1—C10—C9	160.86 (19)	C15—Fe2—C24—C20	−79.75 (19)
N2—N1—C11—C1	−177.6 (2)	N6—N5—C25—C15	−176.7 (2)
N2—N1—C11—C12	1.7 (4)	N6—N5—C25—C26	3.7 (4)
C5—C1—C11—N1	−12.9 (3)	C16—C15—C25—N5	7.8 (4)
C2—C1—C11—N1	165.4 (2)	C19—C15—C25—N5	−172.6 (2)
Fe1—C1—C11—N1	−103.9 (3)	Fe2—C15—C25—N5	97.6 (3)
C5—C1—C11—C12	167.7 (2)	C16—C15—C25—C26	−172.5 (2)
C2—C1—C11—C12	−14.0 (3)	C19—C15—C25—C26	7.1 (4)
Fe1—C1—C11—C12	76.7 (3)	Fe2—C15—C25—C26	−82.8 (3)
N4—N3—C13—C6	175.0 (2)	N8—N7—C27—C20	176.1 (3)
N4—N3—C13—C14	−4.6 (4)	N8—N7—C27—C28	−3.1 (5)
C7—C6—C13—N3	−10.5 (3)	C24—C20—C27—N7	14.5 (4)
C10—C6—C13—N3	171.1 (2)	C21—C20—C27—N7	−165.5 (3)
Fe1—C6—C13—N3	−100.2 (3)	Fe2—C20—C27—N7	103.8 (3)
C7—C6—C13—C14	169.2 (2)	C24—C20—C27—C28	−166.3 (3)
C10—C6—C13—C14	−9.2 (3)	C21—C20—C27—C28	13.8 (4)
Fe1—C6—C13—C14	79.5 (3)	Fe2—C20—C27—C28	−77.0 (3)

### Hydrogen-bond geometry (Å, º)

**Table:**

*D*—H···*A*	*D*—H	H···*A*	*D*···*A*	*D*—H···*A*
N2—H2*A*···N7^i^	0.90	2.53	3.287 (5)	142
N4—H4*A*···N5^ii^	0.90	2.29	3.137 (4)	157
N4—H4*B*···N2^iii^	0.90	2.61	3.421 (4)	150
N6—H6*A*···N3^iv^	0.90	2.24	3.073 (4)	154
N8—H8*B*···N1^v^	0.90	2.60	3.497 (5)	178

Symmetry codes: (i) −*x*+1/2, *y*−1/2, *z*+1/2; (ii) *x*−1, *y*, *z*; (iii) *x*−1/2, −*y*+1/2, *z*; (iv) *x*+1, *y*, *z*; (v) −*x*+1, −*y*+1, *z*−1/2.
